# Occurrence of Melamine and Cyanuric Acid in Breast Milk: A Risk Assessment for Infants Aged 0–6 Months

**DOI:** 10.1002/fsn3.70831

**Published:** 2025-08-25

**Authors:** Leila Nezamoleslami, Arash Ghoorchian, Nishtman Zamani, Tayyebeh Madrakian, Amin Sharifi, Arezo Kavee, Mitra Javdan, Vahid Ghasemzadeh‐Mohammadi

**Affiliations:** ^1^ Department of Nutrition and Food Hygiene, School of Medicine Hamadan University of Medical Sciences Hamadan Iran; ^2^ Department of Chemistry Research Center for Development of Advanced Technologies Tehran Iran; ^3^ Department of Analytical Chemistry, Faculty of Chemistry and Petroleum Sciences Bu‐Ali Sina University Hamedan Iran; ^4^ Deputy Minister of Health Hamadan University of Medical Sciences Hamadan Iran

## Abstract

Melamine (MEL) and cyanuric acid (CYA) are contaminants that can enter the human body through dietary sources, raising significant toxicological concerns, particularly for infants. MEL undergoes minimal biotransformation in the human body. While breast milk is widely recognized as the optimal source of nutrition for infants, it may also serve as a potential route for the transmission of contaminants. This study aimed to quantify the concentrations of MEL and CYA in breast milk and assess the associated non‐carcinogenic toxicity risks for infants aged 0–6 months. A total of 100 mothers from Hamadan, Iran, who exclusively breastfed their infants aged 15–150 days, were included. Breast milk samples, ranging from 10 to 50 mL, were manually expressed. The results revealed that 77% of the samples contained MEL, while 84% contained CYA, both exceeding the detection limits. The average concentrations of MEL and CYA were 730 ± 26 and 400 ± 38 ng mL^−1^, respectively. Risk assessment indicated that none of the infant groups exhibited a hazard quotient (HQ) or cumulative risk (hazard index) from MEL and CYA exposure. According to Monte Carlo simulation, the 95th percentile of HQ for MEL and CYA in breast milk were 0.00561 and 0.0000154, respectively, both well below the safety threshold (HQ < 1). These findings suggest that breast milk consumption by infants up to 6 months of age in Hamadan does not pose a significant risk in terms of MEL and CYA exposure.

## Graphical abstract

The results revealed that 77% of the samples contained MEL, while 84% contained CYA above the detection limit. The mean concentrations of MEL and CYA were 0.73 ± 0.026 mg L^−1^ and 0.4 ± 0.038 mg L^−1^, respectively. Risk assessment indicated that none of the infant groups faced a hazard quotient (HQ) or cumulative risk (hazard index) from MEL and CYA exposure. Based on Monte Carlo simulation results, the 95th percentile of the HQ for MEL and CYA in breast milk was 0.00561 and 0.0000154, respectively, both well below the safety threshold (HQ < 1).

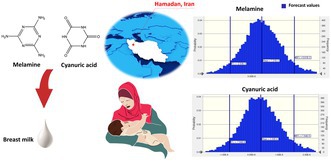

## 1 Introduction

Melamine (MEL, C_3_H_6_N_6_) is a nitrogen‐rich organic compound, classified as a urea derivative, that typically appears as a white powder. When combined with cyanuric acid (CYA, C_3_H_3_N_3_O_3_), it forms insoluble MEL cyanurate crystals, which can accumulate in the renal tubules, potentially leading to kidney stones and other complications (Tyan et al. 2009). Initially, MEL was explored as a potential non‐protein nitrogen (NPN) additive in plant fertilizers and cattle feed due to its high nitrogen content (66.6% by mass). However, its slow and incomplete hydrolysis led to the discontinuation of these applications (Dorne et al. 2013). Subsequently, MEL was illegally added to various food products, including dairy items, confectionery, baked goods, chocolate, wheat flour, pet food, nutritional supplements, and eggs, to artificially elevate protein measurements. Particularly concerning was its illegal incorporation into infant formula to increase its protein content falsely (Wen et al. 2016). In December 2008, the World Health Organization (WHO) convened an expert panel to evaluate the toxicological risks associated with MEL exposure (WHO 2009). Following this assessment, a tolerable daily intake (TDI) of 0.2 mg/kg body weight was established for MEL. However, due to insufficient data, no TDI was determined for CYA. In a separate contamination ncident in 2007, the European Food Safety Authority (EFSA) adopted a higher TDI of 0.5 mg/kg body weight for MEL (EFSA 2008). CYA may occur as a degradation product of MEL or as a microbial metabolite (Tittlemier 2010). Several studies have documented the presence of MEL and CYA in various food matrices. For example, one study reported that the concentration of CYA in dairy products, including milk, was four times higher than that of MEL (Zhu and Kannan 2019b). Although breast milk is considered the optimal source of nutrition for newborns, studies have demonstrated its potential to expose infants to chemical contaminants. Animal model studies have shown that MEL has the potential for both gestational and lactational transfer (Baynes et al. 2010). A 2020 study in Turkey found that MEL was present in 32.2% and 24.4% of first and second breast milk samples, respectively, with 16.7% of women testing positive in both samples (Yalçin et al. 2020). Similarly, a US study found that MEL and CYA were detected in 78% and 95% of samples from children aged 4 months to 8 years (Sathyanarayana et al. 2019). In Canada, researchers detected MEL and CYA in nearly all infant formula products, with maximum concentrations of 0.32 and 0.45 mg/kg, respectively (Braekevelt et al. 2011). In China, the mean urinary concentrations of MEL and CYA in adults were reported as 1.51 and 5.86 μg/L, respectively (Guo et al. 2020). Additionally, a study in Sri Lanka identified MEL in 22 dried milk products, with concentrations ranging from 0.33 to 0.96 mg/kg. High‐fat infant formula contained MEL within the range of 0.39 to 0.84 mg/kg (Strashnov et al. 2021). In a study conducted in Turkey in 2025, the mean concentration of 0.0323 ± 0.0167 μg/mL of MEL was detected in all breast milk samples (Cinkilli Aktağ et al. 2025). Therefore, the objective of this study was: (i) to quantify MEL and CYA levels in breast milk collected from lactating mothers in Hamadan, Iran, and (ii) to assess the non‐carcinogenic risk to exclusively breastfed infants aged 1–6 months.

## 2 Materials and Methods

### 2.1 Chemicals and Reagents

The MEL (CAS 108‐78‐1) and CYA (CAS 108‐80‐5) were purchased from Santa Cruz Biotechnology Inc., and Methanol (purity: 99.96%). They were sourced from Zakaria Jahrom Co. (Jahrom, Iran). Additional chemicals, including KH_2_PO_4_/K_2_HPO_4_ (phosphate buffer, 2.3:7.7, pH = 7.3), were procured from Mojallali Chemical Complex Co.

### 2.2 Gathering Samples

One hundred mothers from Hamadan, Iran, with infants aged 15–150 days who were exclusively breastfed, participated in this study. Key information, such as the infants' age and nutritional status, was collected. The infants' weights were measured, and 10–50 mL of breast milk was collected using manual expression. The samples were transported to the laboratory in plastic bottles within ice‐cooled containers and stored at 4°C for up to 48 h until analysis.

### 2.3 Determination of MEL and CYA

MEL and CYA were quantified following the method of (Venkatasami and Sowa 2010) with some modifications. Briefly, 5 mL of a 1000 ppm MEL and CYA standard solution was added to 5 mL of the milk sample, and the mixture was diluted with 50% methanol to a final volume of 50 mL. The mixture was then subjected to ultrasonication (vCLEAN 1 l6, Backer) for 30 min. The material was centrifuged (D‐7200 Tuttlingen, Hettich) for 10 min at 5000 rpm after cooling. A 50 mL tube was then filled with 10 mL of the supernatant, which had been diluted with 50% methanol. A 0.45 μm Teflon membrane filter was used to filter the solution, and 3 mL of the filtrate was then added to the high‐performance liquid chromatography (HPLC) apparatus for examination.

### 2.4 HPLC Condition

An HPLC system (Knauer, Berlin, Germany) equipped with a UV detector (Model K‐2600, Knauer Smartline, Germany) and a K‐1001 piston pump was used for chromatographic analysis. A reversed‐phase C18 column (4.6 mm × 250 mm, 5 μm particle size) was employed to separate MEL and CYA. To simultaneously determine MEL and CYA, the method of Sun et al. was applied (Sun et al. 2011). The mobile phase consisted of 0.1 mM KH_2_PO_4_‐K_2_HPO_4_ (2.3:7.7) buffer solution (pH 7.3) and methanol (75:25, v/v). The sample injection volume was 20 μL, with a mobile phase flow rate of 0.5 mL/min. A gradient elution program was optimized as follows: 0–3 min, 0% methanol (for CYA separation); 4–16 min, 25% methanol (for MEL separation). The detection wavelengths were 214 nm for CYA and 204 nm for MEL and CYA.

### 2.5 Deterministic Risk Assessment

The estimated daily intake (EDI) was calculated using the following formula:

$$\mathrm{EDI}=\sum C_{i}\times {\mathrm{FIR}}_{i}$$

Where:

- EDI (ng/kg body weight per day) represents the estimated intake for infants less than 6 months of age.
- Cᵢ (ng/mL) is the measured concentration of MEL or CYA in breast milk samples.
- FIRᵢ (mL/kg body weight/day) is the daily food intake rate (Smith 2013).
- The risks of exposure to MEL and its CYA were estimated as Hazard Quotient (HQ), as shown in the following equation:

$$\mathrm{HQ}=\frac{\mathrm{EDI}}{\mathrm{TDI}}$$

Where TDI refers to the tolerable daily intake (ng/kg body weight) of MEL or CYA, this study used the most conservative TDI values, i.e., 3150 ng/kg bw/day for MEL and 2500 ng/kg bw/day for CYA (Choi et al. 2010).

### 2.6 Hazard Index (HI)

The (HI) was calculated to assess the cumulative non‐carcinogenic risk associated with exposure to MEL and CYA. HI was determined using the following formula:

$$\mathrm{HI}=\sum_{k=1}^{n}\mathrm{HQ}=\text{HQmel}+\text{HQcya}$$

- If HI > 1, the exposed population may face non‐carcinogenic health risks.
- If HI < 1, no significant health hazards are anticipated (U. EPA 2011).

### 2.7 Probabilistic Risk Assessment (PRA)

A probabilistic approach was employed to account for uncertainties in risk estimation. The Monte Carlo Simulation (MCS) was used to estimate MEL and CYA exposure levels in different subpopulations. Monte Carlo simulations were performed using Crystal Ball software (Oracle). To reflect realistic uncertainty in input variables, the concentrations of MEL and CYA were modeled using a normal distribution, given their symmetric spread around the mean. In contrast, log‐normal distributions were applied to other variables, such as infants' body weight (bw) and FIR, due to their positively skewed nature and variability in biological populations. The Kruskal‐Wallis test was used to assess differences in consumption levels across gender and age groups. The chi‐square test was used to evaluate the percentage of children who exceeded the TDI for MEL and CYA. The results of the Monte Carlo simulation were based on 10,000 iterations, and the 95th percentile of the HQ distribution was used to assess the risk.

### 2.8 Demographic Questionnaire and Data Analysis

A demographic questionnaire was designed to collect information on mothers' age, weight, smoking habits, cosmetic use, employment status, and infants' age and weight. The data were analyzed using IBM SPSS Statistics 26 (Armonk, New York, USA). Numerical data were summarized using descriptive statistics, and categorical variables were reported as frequency distributions. To better understand exposure based on infant characteristics, the mothers were divided into three groups:

1. Infants aged 0–2 months.
2. Infants aged 2–4 months.
3. Infants aged 4–6 months.

Categorical variables were summarized using frequency distributions. Numerical variables were analyzed with descriptive statistics. The Mann–Whitney *U* test and Kruskal–Wallis test were used to assess differences in consumption levels across gender and age groups. The chi‐square test was used to evaluate the percentage of children exceeding the TDI for MEL and CYA. The exact binomial test was used to calculate percentage values with a 95% confidence interval (CI). A *p*‐value < 0.05 was considered statistically significant.

## 3 Results and Discussion

### 3.1 Method Validation and Concentration of Melamine and Cyanuric Acid

The linear regression correlation coefficients for MEL and CYA exceeded 0.995, indicating a strong linear relationship. The method's repeatability was confirmed by the precision, represented by the relative standard deviation (RSD), which was 13% for MEL and 14% for CYA. Signal‐to‐noise ratios of 3:1 and 10:1 were used to determine the limits of detection (LOD) and quantification (LOQ), respectively. The linearity ranges were 100–10,000 ng mL^−1^ for both MEL and CYA. As presented in Table 1, the peak concentrations of MEL and CYA were 1610 ng mL^−1^ and 1420 ng mL^−1^, respectively, while the minimum concentrations were 670 ng mL^−1^ and 670 ng mL^−1^ respectively. Overall, 77% of the samples contained MEL, whereas 84% contained CYA above the LOD threshold. Previous investigations have reported the LOD for MEL and CYA in breast milk as 0.02 and 0.01 ng mL^−1^, respectively, and the LOQ as 0.06 and 0.03 ng mL^−1^ (Zhu and Kannan 2019c) and for MEL as 10.62 and 41.55 ng L^−1^ (Yurdakok et al. 2014). The LOD and LOQ in infant formula values were reported as 100 ng mL^−1^ and 200 ng mL^−1^, respectively (Venkatasami and Sowa 2010), and 1000 and 3000 ng mL^−1^, respectively (Mazaheri et al. 2024). While using simplified procedures without complicated purification processes can save time and cost benefits, it can also lead to higher RSD, LOD, and LOQ values. Depending on the method used, the matrix effect is likely caused by the remaining proteins in the final solution before HPLC. In line with our studies, previous research has also been conducted in various countries. For instance, Yurdakok and coworkers detected MEL in 16 out of 77 breast milk samples, with concentrations ranging from 0.0109 to 0.0764 ng mL^−1^ (Yurdakok et al. 2014). Similarly, Yalçin et al. (2020) reported that 40% of breast milk samples from women in Şanlıurfa, Turkey, contained MEL and/or CYA at levels of ≥ 200 ng mL^−1^ and ≥ 27 ng mL^−1^, respectively. Furthermore, a U.S. study by Zhu and Kannan (2019c) indicated that 89% of breast milk samples contained MEL, while 100% had detectable levels of CYA. According to Iranian research, the MEL content in milk powders varied from 1 to 4 ng mL^−1^, whereas it ranged from 1 to 95 ng mL^−1^ in baby formula samples (Mazaheri et al. 2024). Additionally, research conducted by Ghanati et al. (2023) in Iran found that the MEL content of packaged milk varied, with levels of 790 ± 39.8 ng mL^−1^ in carton packs, 50.7 ± 13 ng mL^−1^ in plastic packaging, and 57.7 ± 24.54 ng mL^−1^ in polyethylene bags. In a 2024 study conducted in Nigeria, the concentration of MEL in milk was 57.6 ± 18.9 and 930.3 ± 379.9 ng mg^−1^ (Oyedeji et al. 2024). In a study conducted in Iran, the concentration of MEL in infant formula was found to be 730 ± 710 ng mg^−1^ (Maleki et al. 2018). In a 2021 study by Abedini and colleagues, MEL was detected in approximately 94% of imported chocolate samples and about 77% of Iranian chocolates (Abedini et al. 2021). The concentration of MEL in eggs was 1100–28,700 ng mg^−1^ (Bai et al. 2010). These findings highlight the presence of MEL in various food products, as reported by Abedini et al. (2023). Consequently, MEL may be present in the body either in its natural state or as derivatives, including CYA. The widespread use of MEL in various materials that mothers utilize daily further contributes to its presence in breast milk (Lütjens et al. 2023). One study indicated that children in the MEL‐related bladder stones group exhibited delayed growth compared to the control group, with significant differences in height and weight (Yang et al. 2013). The bivariate association between the MEL/CYA concentrations and the initially chosen variables is displayed in Table 2. Overall, there was no significant relationship between maternal factors and the amounts of MEL or CYA in breast milk (*p* > 0.05), which is in line with prior research findings (Yalçin et al. 2020; Yesildemir et al. 2023; Zhu and Kannan 2019c). The small sample size and the analytical method may have limited the ability to detect potential influencing factors, despite the possibility that certain conditions could affect MEL and CYA levels in breast milk.

**TABLE 1:** Method performance parameters and concentration values of MEL and CYA in samples.

	MEL	CYA
LOD value (ng. mL^−1^)	90	100
LOQ value (ng. mL^−1^)	270	310
Recovery %	97–102	93–110
Linearity range (μg/mL^−1^)	0.1–10	0.1–10
RSD	13%	14%
Samples < LOD	23	16
Mean ± SD in all samples	730 ± 26	400 ± 38
Range of toxicant in samples (ng/mL^−1^)	(670–1610)	(310–1420)

**TABLE 2:** Demographic characteristics of the mothers related to melamine (MEL) and cyanuric acid (CYA) in breast milk.

		MEL (ng. mL^−1^)				CYA (ng. mL^−1^)		
		*N*	Mean	± SD	*p*	Mean	± SD	*p*
Age	< 31	12	600	15	0.602	400	10	0.662
	> 31	18	900	17		500	10	
Pre‐BMI	< 25	16	400	9	0.492	100	20	0.380
	> 25	29	700	14		500	10	
Education	Under diploma	22	500	11	0.625	300	80	0.545
	Diploma and higher	28	700	13		400	80	
Income level	Unknown	17	500	12		400	90	
	50 million Rial	20	400	50	0.419	300	40	0.587
	More than 5 million Rial	13	200	18		500	10	

### 3.2 Calculating Melamine and Cyanuric Acid Toxicity in Breastfed Babies

All infants in this study were exclusively breastfed and were categorized into three age groups: 0–2 months, 2–4 months, and 4–6 months (Table 3). The EDI of both CYA and MEL was highest in the 2–4 months group, followed by the 0–2 months group. Because the concentration of MEL in the 2–4 months group was higher than in the other groups, the reason for the difference in MEL levels among different age groups may be due to the level of maternal exposure to MEL, which was highest in the 2–4 months group. Among other factors, we can point to the weight of the infants and the amount of milk they consume, which affects FIR. Studies have shown that the concentrations of MEL and CYA in human urine remain relatively constant over several weeks (Zhu and Kannan 2019a). Continuous exposure and rapid excretion of MEL and CYA account for the lack of a significant association between maternal and infant characteristics and measured concentrations in breast milk. Although some factors may influence the concentrations of MEL and CYA in breast milk, the small sample size prevented further interpretation of the data (Zhu and Kannan 2019c). Another study calculated MEL exposure in breastfed infants at 0.12 ng mg^−1^ bw/day and 0.24 ng mg^−1^ bw/day (Yesildemir et al. 2023). However, the reported EDI via infant formula consumption was significantly higher than that via breastfeeding (Tittlemier et al. 2009; Zhu and Kannan 2018). As shown in Table 3, the median HQs estimated for MEL and CYA across different infant age groups were all below 1, indicating no risk of non‐carcinogenic toxicity. The average HQ calculated for MEL and CYA for different age groups of infants was below 1, indicating minimal risk to American infants (Zhu and Kannan 2019c). The HI, which represents the combined risk of MEL and CYA exposure from breast milk, was also below 1, suggesting that infants are not at risk of adverse health effects from these contaminants. However, non‐breastfed infants may face a higher non‐carcinogenic risk, as evidenced by a study reporting HQs of 2.2280, 0.9444, 0.5714, and 0.6714 for MEL in infant formula (Abedini et al. 2023).

**TABLE 3:** Estimated daily intake (EDI; ng/kg body weight per day), risk factor (HQ) and HI of cyanuric acid and melamine through breast milk feeding.

Age (by month)	*N*	EDI (cyanuric acid)	HQ (cyanuric acid)	EDI (melamine)	HQ (melamine)	HI
(0–2)	20	32.10	0.01	72.60	0.02	0.03
(2–4)	15	125.98	0.05	172.46	0.05	0.10
(4–6)	15	26.56	0.01	35.87	0.01	0.02

### 3.3 Probabilistic Risk Assessment

**FIGURE 1:**
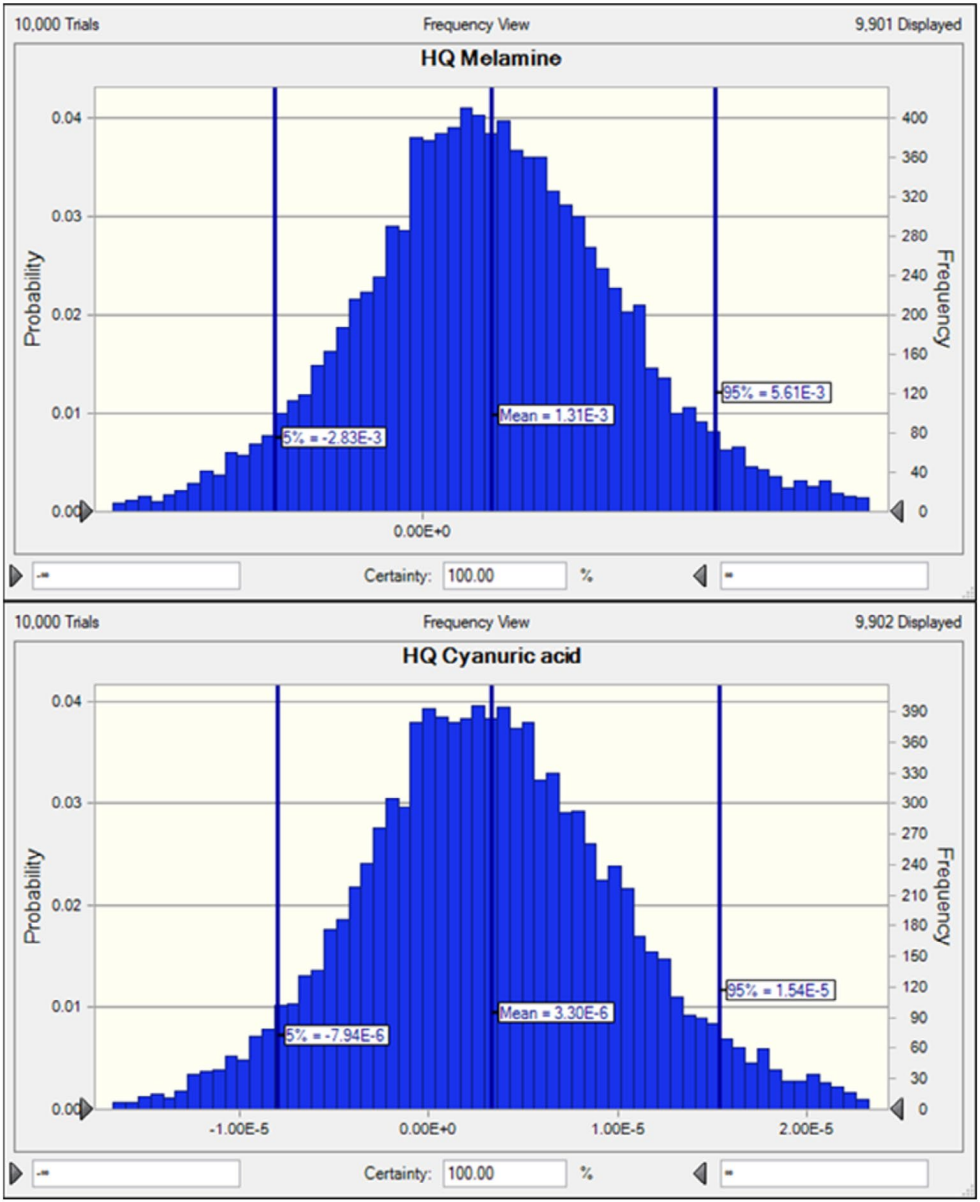
Probabilistic non‐carcinogenic risk assessment of MEL and CYA in breast milk in infants according to Monte Carlo simulation.

Numerous uncertainties may influence the risk assessment process. Utilizing point estimates to evaluate the health risk of contaminant exposure introduces significant uncertainty. Therefore, Monte Carlo simulation (MCS) was employed to reduce uncertainties (Doménech and Martorell 2024). This approach involves repeatedly selecting random values from the probability distribution of multiple inputs to calculate a risk probability distribution. The MCS technique enhances the precision of pollutant exposure risk assessment by incorporating uncertainties, as proposed by the USEPA. The simulation was conducted based on predefined factors, including concentration, dosage, and weight, and was performed over 10,000 iterations. The EDI of MEL and CYA in milk was 0.00891 and 0.00571 ng mg^−1^ bw/day, respectively. The 95th percentile of the HQ distribution is illustrated in Figure 1. The mean cumulative probability (P) of 95% of milk consumption in infants indicates that the HQ for non‐carcinogenic risk assessment remains below the safe threshold recommended by the USEPA (U.S. EPA 2011). To assess the influence of uncertainty on risk outcomes, a probabilistic model was developed that incorporates distributions for both MEL and CYA concentrations, as well as intake and body weight. The simulation outputs showed that variations in contaminant concentration and FIR were the most influential factors affecting the estimated risk. Despite this, the 95th percentile HQs for MEL (0.00561) and CYA (0.0000154) remained well below the safety threshold (HQ < 1). These results indicate that, although uncertainty exists, the cumulative effect does not significantly alter the conclusion that exposure to both MEL and CYA in breast milk poses a minimal health risk. Beyond its presence in food products, MEL is widely utilized in industrial applications, including the production of plastics, insulation materials, cleaning agents, and flame retardants (Lütjens et al. 2023). CYA, a structurally related analog of MEL, is commonly found in swimming pool water as a byproduct of dichloroisocyanurate decomposition, which is used as a water disinfectant. Additionally, it serves as a chlorine stabilizer and is employed in disinfectants and bleach (Bischoff 2022). In the probabilistic risk assessment, the distributional assumptions and variability in input parameters were found to significantly influence the risk estimates. Both MEL and CYA concentrations in breast milk were modeled using normal distributions, while infant body weight and milk intake rate (FIR) followed log‐normal distributions to reflect biological variation. The results indicated that changes in FIR and contaminant concentration had the greatest impact on the estimated HQ. Although the 95th percentile HQ values for both compounds remained well below the safety threshold (HQ < 1), the probabilistic modeling revealed a wider spread in risk estimates compared to deterministic analysis. This highlights the importance of incorporating uncertainty in exposure modeling to better capture potential variations and provide a more realistic and transparent risk characterization.

## 4 Conclusions

MEL contamination remains a major global health issue. Women can be exposed to both MEL and CYA throughout their lives, particularly during lactation, which leads to the transfer of these contaminants to infants via breast milk. Around 50% of the samples showed measurable levels of these chemicals, above the LOQ. Factors like age, income, education, and pre‐BMI had no significant impact on MEL and CYA concentrations in breast milk. After analyzing the non‐carcinogenic risks using both deterministic and probabilistic approaches, none of the infant groups in this study was found to be at risk. Additionally, the (HI) showed no indication of any cumulative risk associated with these contaminants. These results highlight the importance of continued monitoring of MEL and CYA levels in food products to reduce potential health risks. Limitations of the study included the lack of cooperation from some mothers in providing samples and completing the questionnaire, as well as the small sample size. Finally, it is recommended to establish regulatory limits for MEL and CYA in breast milk and other food products.

## Author Contributions

**Leila Nezamoleslami:** investigation (equal). **Arash Ghoorchian:** validation (lead), writing – original draft (supporting), writing – review and editing (supporting). **Nishtman Zamani:** writing – original draft (supporting). **Tayyebeh Madrakian:** conceptualization (equal), methodology (equal). **Amin Sharifi:** investigation (equal). **Arezo Kavee:** data curation (equal). **Mitra Javdan:** data curation (equal). **Vahid Ghasemzadeh‐Mohammadi:** conceptualization (lead), methodology (equal), writing – original draft (equal), writing – review and editing (equal).

## Ethics Statement

IR.UMSHA.REC.1399.495.

## Consent

All participants granted informed consent.

## Conflicts of Interest

The authors declare no conflicts of interest.

## Data Availability

The data that support the findings of this study are available on request from the corresponding author. The data are not publicly available due to privacy or ethical restrictions.
